# Retraction Note: Acid-modified CNT/Zinc Oxide nanowires based high performance broadband photodetector

**DOI:** 10.1038/s41598-025-09235-0

**Published:** 2025-07-01

**Authors:** K. Moatemsu Aier, Jay Chandra Dhar

**Affiliations:** Department of Electronics and Communication Engineering, National Institute of Technology Nagaland, Chumukedima, Nagaland 797103 India

Retraction of: *Scientific Reports* 10.1038/s41598-023-30426-0, published online 23 February 2023

The Editors have retracted this Article.

After publication, concerns were raised regarding high image similarities across publications from the same Authors and data integrity issues. Specifically:

- There appear to be irregularities in the determination of the peak height of the elements for the EDX spectra in Figures 2C-D and Figure 3D;
- R v/s power density profile in Figures 11C-D seems to present unusual gaps in the D* graph line.

Upon request, the Authors provided raw data for the EDX spectra in Figures 2C-D and Figue 3D. These data appear discrepant with the published results. The raw data for the R v/s power density profile in Figures 11C-D did not contain metadata that would allow for verification of the veracity of the data. Additionally, the Authors were unable to provide a satisfactory physical explanation concerning the image irregularity of Figures 11C-D. The Editors therefore no longer have confidence in the presented data.

The Authors disagree with this retraction.

